# Missing data imputation of climate time series: A review

**DOI:** 10.1016/j.mex.2025.103455

**Published:** 2025-06-19

**Authors:** Lizette Elena Alejo-Sanchez, Aldo Márquez-Grajales, Fernando Salas-Martínez, Anilu Franco-Arcega, Virgilio López-Morales, Otilio Arturo Acevedo-Sandoval, César Abelardo González-Ramírez, Ramiro Villegas-Vega

**Affiliations:** a Área Académica de Computación y Electrónica, Instituto de Ciencias Básicas e Ingeniería, Universidad Autónoma del Estado de Hidalgo, Carr. Pachuca-Tulancingo km. 4.5, Mineral de la Reforma, 42184 Hidalgo, Mexico; b Área Académica de Química, Instituto de Ciencias Básicas e Ingeniería, Universidad Autónoma del Estado de Hidalgo, Carr. Pachuca-Tulancingo km. 4.5, Mineral de la Reforma, 42184 Hidalgo, Mexico; c Artificial Intelligence Research Institute, University of Veracruz, Campus Sur Paseo Lote II, Sección Segunda N° 112, Nuevo Xalapa, 91097 Veracruz, Mexico

**Keywords:** Climate time series, Missing data, Imputation, Machine learning, Deep learning, Imputation techniques for handling missing data

## Abstract

Missing data in climate time series is a significant problem because it complicates the monitoring and prediction of climatic phenomena. The primary objective of this research document is to describe the most relevant imputation methods for missing data in the climate context over the last decade. Results reveal a superior concentration of documents on the use of imputation methods for climate time series in Asia and Europe, with notable examples from Malaysia, China, and Italy. Meanwhile, Brazil and Australia were the countries with a high number of research in America and Oceania. Moreover, temperature and precipitation were the most frequently employed climate variables. Regarding the information source, the monitoring networks were the most commonly used source for extracting data in almost all the research. On the other hand, methods such as mean techniques, simple and multiple linear regression, interpolation, and Principal Component Analysis (PCA) were the conventional statistical techniques used for imputing missing data. Furthermore, artificial neural networks demonstrated the ability to identify complex patterns in the data. Finally, Generative Adversarial Networks excel over other deep learning methods in the imputation of missing climate data.

## Review Highlights

• This review presents the most used techniques for the imputation of missing data on climate time series.
• Moreover, this document highlights the countries that have produced significant research on this problem.
• Finally, this review encourages new research lines for imputing climate time series.

## Graphical abstract

**Figure:**
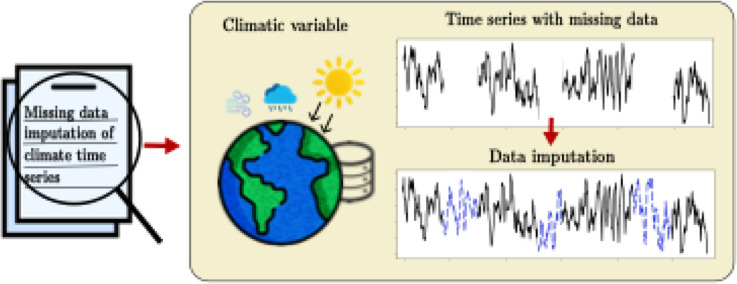

Specifications table

**Table:** 

Subject area:	Computer Science
More specific subject area:	Artificial Intelligence
Name of the reviewed methodology:	Imputation techniques for handling missing data
Keywords:	Climate time series; missing data; imputation; machine learning; deep learning
Resource availability:	Not applicable
Review question:	What is the region/country when there are more publications about missing data?
	Which conventional, machine learning-based, and deep learning-based methods are used to imputation missing data in climate time series?

## Background

Nowadays, the worldwide constant climate changes have generated several natural extreme phenomena. Therefore, most countries emphasize the importance of climate information For example, organizations such as the United Nations Framework Convention on Climate Change (UNFCCC) propose international policies related to climate change studies. The UNFCCC, in its Paris Agreement [1], emphasized the importance of strengthening scientific knowledge about climate, including research, observation, and systematic monitoring, as well as the early warning of climate phenomena. Consequently, the member countries of this institution must ensure climate monitoring at the local and regional levels [1].

Additionally, in the same year, the Sustainable Development Goals were established, incorporating Goal 13, which focuses on climate actions, as part of these goals. Goal 13 specifies improving education, sensitization, and human and institutional capacity for climate change mitigation, adaptation, effects reduction, and early warning [2]. On the other hand, Goals 2 (zero hunger), 6 (clean water and sanitation), 11 (sustainable cities and communities), 14 (life below water), and 15 (life and land) are limited to meet their purposes, due to the climate change impact on their interests [2]. Hence, the importance of generating and recording climate information about climate monitoring, extreme and slow-evolving phenomena is standing out, as these climatic events have a direct impact on human activities and the environment.

However, this task requires a high-quality technological infrastructure for adequate and consistent storage, which necessitates dedication, effort, and substantial economic resources. Otherwise, the data may be altered during storage or not recorded at all. This issue poses a challenge for most countries, complicating the implementation of warning systems and leading in datasets with significant data loss.

Missing data in climatic datasets impede the monitoring and prediction of natural phenomena and their intensities. Data imputation can face this problem through various techniques, including conventional (statistics) and artificial intelligence methods. As a consequence, the purpose of this article is to review the state-of-the-art techniques employed for imputing climate missing data, as well as the countries where these techniques are mainly applied and the types of climate variables used.

In the literature, several reviews have been proposed to analyze the techniques used for handling missing data in time series. Most of them were proposed to analyze missing data strategies in various fields, such as medicine [[3], [4], [5], [6], [7], [8], [9], [10], [11]], biology [12,13], and physics [14], or a general context [[15], [16], [17], [18], [19]]. However, a small portion is focused on climate time series and only one variable [[20], [21], [22], [23]]. Consequently, the main contribution of this review is an exhaustive analysis of imputation methods employed for handling missing data in climate time series. Unlike the reviews proposed in the literature, this manuscript is not restricted to a single climatic variable; rather, those used to describe different climatic phenomena are considered. Moreover, most of the missing data imputation techniques are covered and grouped into conventional (statistical), machine learning, and deep learning techniques. In summary, this review analyzes the methods and contexts in which they are employed to handle missing data in climate time series more broadly.

## Method details

### Search process description

The systematic literature review performed in this manuscript is based on [24], from which six steps were performed to extract the information for the analysis. These steps are described below.

1. *Defining keywords.* The keywords employed to search our data sources were missing data, imputation data, time series, weather, rainfall, temperature, climate, and meteorological. These keywords encompass the general purpose of our manuscript.
2. *Selecting digital library source.* Regarding the digital library source used, four sources were employed to search for the necessary documents in this analysis. These digital libraries were Dimensions (https://app.dimensions.ai/auth/base/landing?redirect=%2Fdiscover), Google Scholar (https://scholar.google.com/), Scopus (https://www.scopus.com/), and ResearchRabbit (https://www.researchrabbit.ai/). Each digital library was selected based on its intuitive interface and the search engine it utilizes.
3. *Defining inclusion and exclusion criteria.* The inclusion and exclusion criteria were defined based on the period, language, source type, and accessibility. The inclusion criterion used for the period was from 2015 to the present, and the exclusion criterion was documents published prior to 2015. Regarding language, the inclusion criterion employed was those documents published in English, excluding those in other languages. The type of sources included were indexed articles, scientific conference papers, and book chapters, excluding pre-print articles and books. Finally, we exclude all unaccessible documents by our institution.
4. *Building digital library search string.* Based on the keywords selected, we defined the search string as "Time series" AND ("weather" OR "Rainfall" OR "Temperature" OR "Climate" OR "Meteorological") AND ("Missing Data" OR "Imputation data"). This search string was employed in each digital library source.
5. *Preprocessing documents.* Once the search is executed, a preprocessing was performed to avoid undesired and duplicated documents. Table 1 shows the number of information sources found and the final number of documents selected.

**Table 1:** Description of the total amount of research found in various digital libraries and the final number after removing duplicate documents.

Database	Documents found	Documents Verified
Dimensions	5	4
Google Scholar	25	19
Scopus	21	20
RabbitResearch	22	17
6. *Extracting principal characteristics.* After selecting the documents that meet the necessary criteria for the review, we identified some features to standardize our analysis. These features included the study area where the information for the series with missing data was collected, the techniques used for the imputation process, the variables used to construct the time series, the advantages and disadvantages of each method, and the findings of each proposal.
7. *Presenting information.* The information is presented based on the features described in the previous step. Figures and tables were employed to facilitate reading comprehension. Moreover, a critical discussion was included in each technique, highlighting the advantages and disadvantages of all methods. Finally, a description of the new research directions detected is presented.

### Study areas analysis

**Fig. 1:**
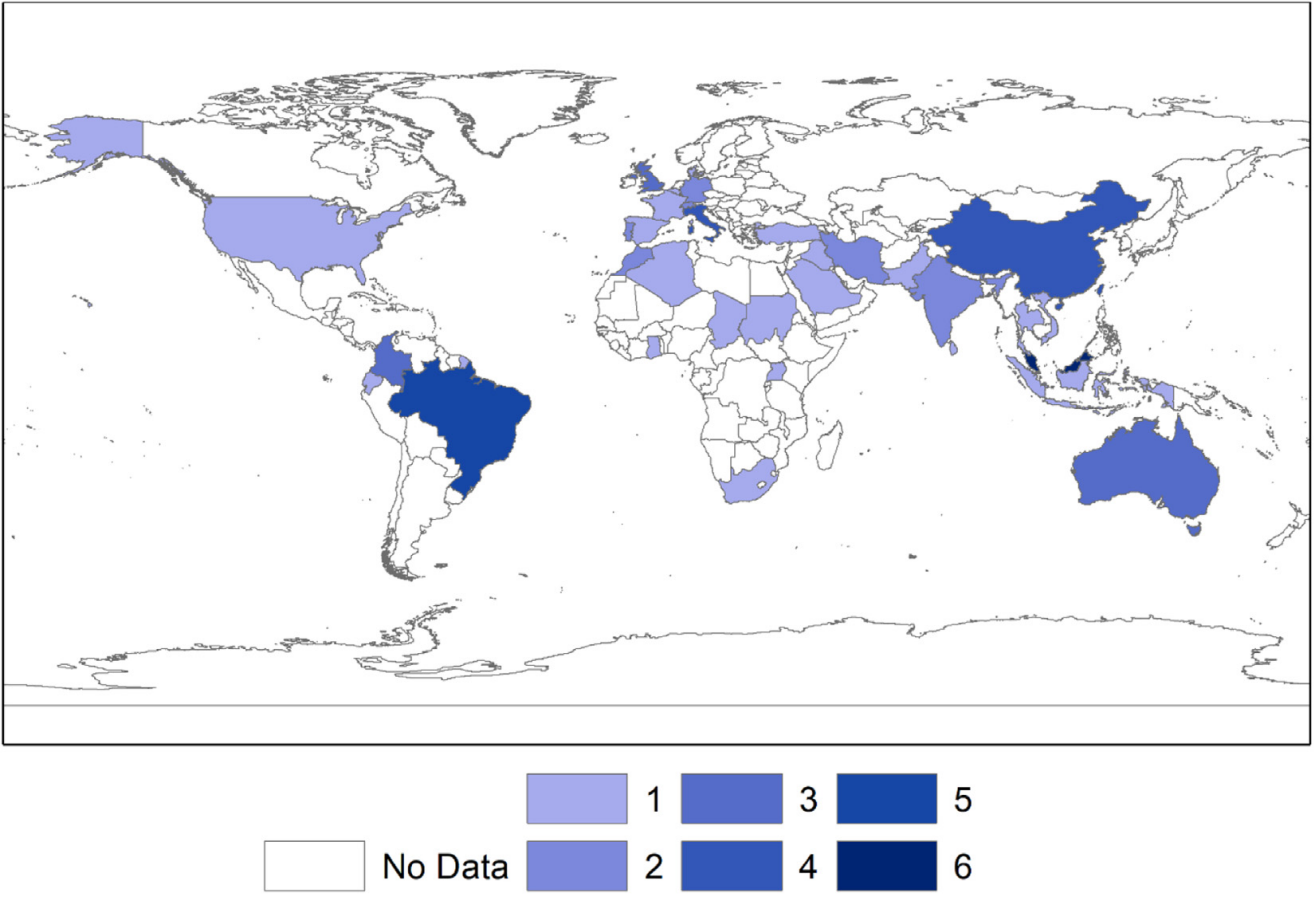
Countries with research on handling missing data in climate time series. The most intense blue color indicates the highest number of publications. Conversely, lighter shades indicate countries with fewer publications.

Regarding the study regions in which time series reconstruction work was performed using meteorological or climatic information, Asia and Europe excel with the most significant number of publications. The country with the most works published in this area was Malaysia [[25], [26], [27], [28], [29], [30]], followed by China [[31], [32], [33], [34]] and Italia [[35], [36], [37], [38]], the latter two with the same number of works. On the other hand, America and Oceania were the continents with the lower number of publications on the imputation of climate missing data. However, Brazil [[39], [40], [41], [42], [43]] and Australia [[44], [45], [46]] were the countries with the highest number of publications, ranking second and fourth among the countries with the highest research production on these continents. Fig. 1 illustrates the distribution of publications by country, and Table 2 lists the number of publications in each country.

**Table 2:** Number of publications per country where a study was conducted.

Country	Frequency
Malaysia	6
Brazil	5
Italy, China	4
Australia, Colombia, United Kingdom	3
India, Morocco, Switzerland, Iran, Germany, Portugal,	2
Iraq, Indonesia, Sri Lanka, Spain, Saudi Arabia, Ghana, Turkey, Denmark, Thailand, Uganda, Chad, Algeria, France, South Africa, Vietnam, United States of America (USA), Belgium, Pakistan, Sudan, Ecuador	1

It is worth noting that only one study on missing data was found in the United States of America (USA), despite the country’s high scientific production. As a developed country and one of the world's largest economies, the USA allocates sufficient economic resources for the integration of efficient climate monitoring networks. Consequently, its climate information must contain a minimum percentage of missing data. For example, this country has an agency dedicated to studying and storing climatic information called the National Oceanic and Atmospheric Administration (NOAA), which is considered a leading reference in the scientific community on climate issues. In the Biden-Harris administration, the Inflation Reduction Act (IRA) was integrated into the US legislative. IRA injected $3.3 billion into NOAA to consolidate its efforts in reducing vulnerability, strengthening resilience to weather and climate events, improving supercomputing capacity and scientific research on climate, oceans, and weather, strengthening the fleet and hurricane-hunting aircraft, and replacing NOAA's obsolete installations [47].

On the other hand, in countries such as Mexico, there is a lack of work related to the reconstruction of time series despite the country experiencing a significant number of meteorological phenomena that affect its population (tropical cyclones, droughts, cold fronts, warm fronts, heat waves, among others) [48]. Moreover, the number of meteorological stations operating in Mexico is decreasing, and the records are not constant, resulting in time series with a large amount of missing data and a decrease in available information [[49], [50], [51]].

Regarding climate variables, the variables most commonly employed for imputing missing data are temperature, precipitation, and relative humidity, with 29, 28, and 15 research works, respectively. The authors emphasize that these variables are crucial for mitigating the impacts of global warming and changing precipitation patterns. On the other hand, the variables least used for imputing missing data are leaf wetness, dew point temperature, evaporation, sunshine duration, latent heat flux, and carbon flux. It is important to mention that, according to the World Meteorological Organization (WMO), the basic instruments for measuring climatic features are thermometers, rain gauges, anemometers, wind vanes, hydrometers, and barometers, which help to measure the behavior of temperature, precipitation, wind, air humidity, and atmospheric pressure, respectively [52,53]. This affirmation aligns with most climate publications, which report data on the mentioned variables through a monitoring process. Table 3 illustrates the relationship between the reconstruction frequency and the climatic variables.

**Table 3:** Frequency of variables used for climate time series imputation.

Variable	Frequency
Room temperature	29
Rainfall	28
Relative humidity	15
Wind	11
Solar radiation	10
Evapotranspiration, Atmospheric pressure	3
Leaf wetness, Dew point temperature, Dew point temperature	2
Evaporation, Duration of sunlight, Heat flux, Carbon flux	1

**Fig. 2:**
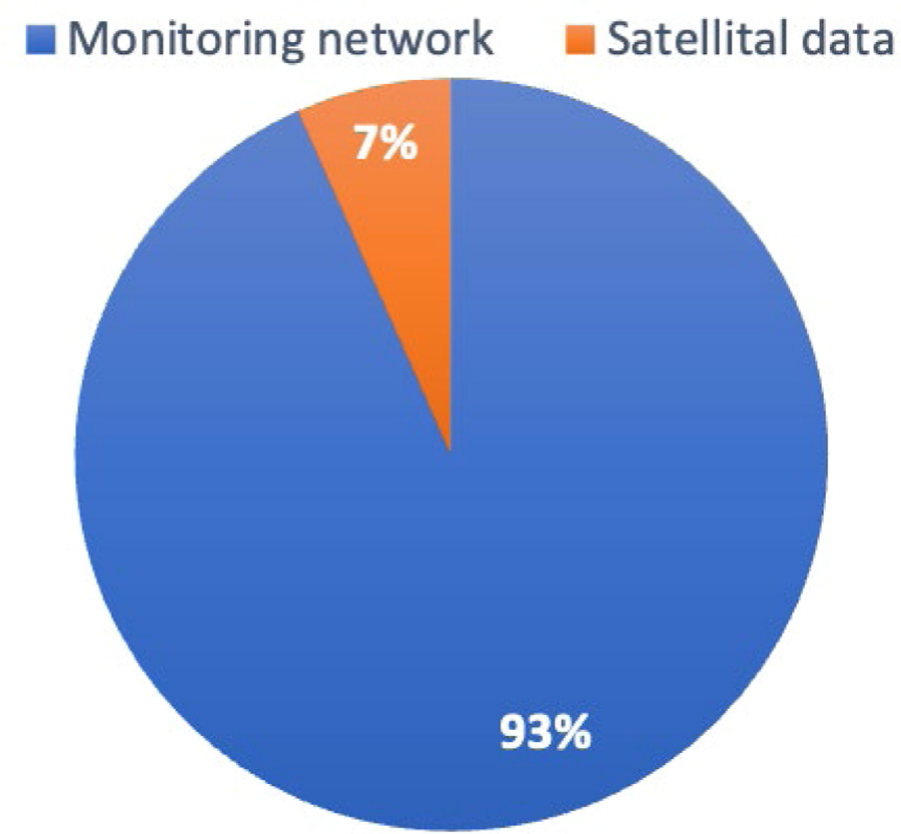
Distribution of the research works according to the data type source. The blue segment indicates the research percentage where imputation was performed using climate monitoring network data. The orange color indicates the research percentage using satellite data.

Moreover, Fig. 2 illustrates the distribution of the research categorized by the data type used for series imputation. This categorization was derived from the information collection process, specifically whether data were collected by a climate monitoring network or a satellite. The findings indicate that 93 % of the information is obtained from monitoring networks. Meanwhile, only 7 % of researchers employ satellite information for this purpose. It is essential to note that studies utilizing satellite data treat the presence of clouds as missing data rather than a the lack of information due to the record error, which is how the monitoring network typically interprets it.

### Conventional methods

**Fig. 3:**
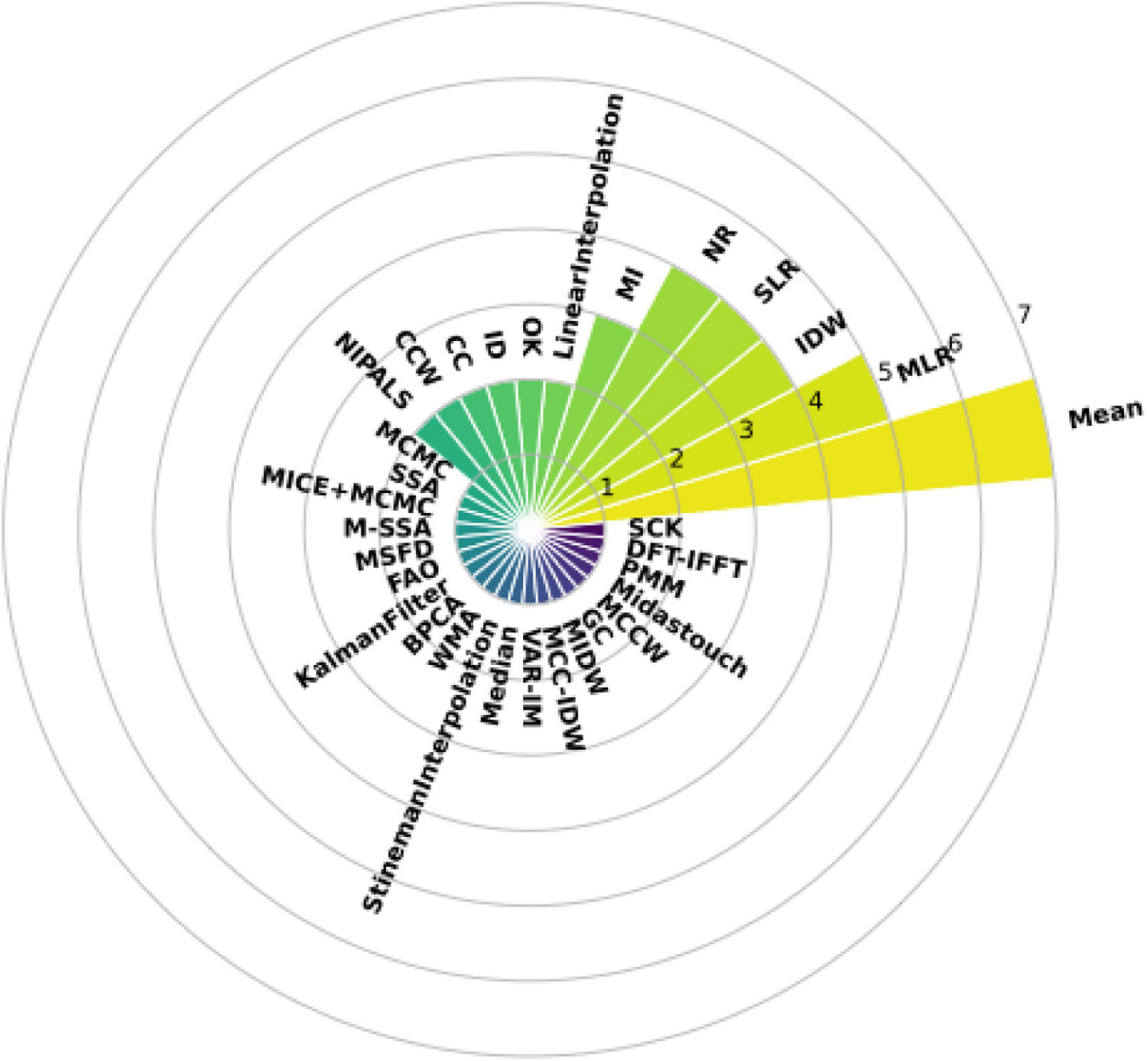
Frequency graph of the most commonly used conventional or statistical methods found in the systematic literature review.

Fig. 3 illustrates the conventional statistical methods employed to impute missing data in climatic time series. The average methods stand out, followed by Multiple Linear Regression (MLR) and Simple Linear Regression (SLR).

Parra-Plazas et al. [54] addressed climatic time series from ten meteorological stations in Cienega, Colombia. Each station transmits data for 14 environmental variables. Different statistical methods were evaluated, including the mean, basic linear regression, the Discrete Fourier Transform (DFT), and Inverse Fast Fourier Transform (IFFT). These techniques were employed to address issues related to outliers and missing data in meteorological variables, including temperature, humidity, and wind speed. For performance evaluation, the Root Mean Square Error (RMSE), similarity index, and Normalized Root Mean Square Error (NRMSE). The DFT-based method proved superior, achieving average errors of 1 % and yielding good results even in multivariate scenarios with high incidence of missing data.

Muhammad et al. [28] focused on evaluating time series trends of four rainfall stations using classical statistical methods in Jeli, Kelantan, Malaysia. The authors employed SLR to initially impute the missing values, given the significant proportion of missing data (up to 41.8 %). Subsequently, they applied the nonparametric Mann-Kendall test and Sen's slope estimator to identify climatic trends. The results showed a statistically significant decreasing trend in a specific season. However, they mention that Kriging, spline interpolation, and machine learning-based approaches can provide more accurate estimates of missing data than simple linear regression.

Maziero et al. [41] analyzed meteorological time series with hourly and daily records from two locations in São Paulo, Brazil. The authors applied statistical methods, such as Predictive Mean Matching (PMM) and Weighted Predictive Mean Matching (midastouch), which are based on Multiple Imputations by Chained Equations (MICE), to variables including global solar radiation, wind speed, temperature, and relative humidity. The prediction accuracy of missing values was determined using the overall NRMSE. The results showed that both methods performed satisfactorily on hourly data because of the higher temporal resolution. However, midastouch especially outperforms PMM due to its lower uncertainty and superior imputation accuracy.

Moreover, Khan and Naeem [55] investigated daily precipitation data for 24 gauging stations in the Upper Indus basin. This region is characterized by mountainous complexity and a sparse network of weather stations. The comparison evaluated eight different statistical methods: Arithmetic Average Method (AAM), Coefficient Correlation Weighting (CCW), Modified Coefficient Correlation Weighting (MCCW), Geographic Coordinates method (GC), Inverse Distance Weighting Method (IDW), Modified Inverse Distance Weighting Method (MIDW), Modified Coefficient Correlation with Inverse Distance Weighting (MCC-IDW), and MLR. The estimation methods were evaluated using four statistical metrics: Mean Absolute Error (MAE), RMSE, Nash-Sutcliffe Efficiency (NSE), and Similarity Index (SI). The MLR method performed best for almost 50 % of the data.

On the other hand, Rizal et al. [56] addressed multivariate imputation using Vector Autoregressive Models (VAR-IM) on daily meteorological data from the Citeko weather station, Indonesia. The variables considered have information on temperature, wind, humidity, precipitation, and solar radiation. This multivariate approach was compared against other traditional univariate methods, including linear interpolation, moving averages, and specialized techniques for seasonal data. Simulations revealed that the proposed modifications to the VAR-IM method benefited accuracy, particularly in scenarios with high proportions of missing data. Besides, the VAR-IM method showed stability in performance accuracy as the proportion of missing data increased, highlighting the usefulness of vector autoregressive models in multivariate time series with strong time dependencies.

Contreras Vargas et al. [57] implemented a methodology based on median imputation for reconstructing time series of air temperature and wind speed variables in the Rosario Islands, Colombian Caribbean. The close point imputation method uses close values to the data order and then selects the median to replace the missing value. This method has the advantage that the replaced data belongs to the original time series data. The results were evaluated using RMSE, which demonstrated an adequate time series imputation of the proposal, highlighting its effectiveness in scenarios with sporadic data losses. However, it was evidenced that, for long consecutive gaps, the imputation method reduces the time series variance.

Duarte et al. [39] compared several statistical and mixed methods for imputing daily and monthly precipitation data with missing data values. Data was extracted from 50 stations in the central-west region of Brazil. The authors evaluated techniques such as SLR and MLR using satellite data from the Global Precipitation Measurement (GPM) mission, processed through the Integrated Multi-satellite Retrievals for GPM (IMERG) algorithm for direct imputation. RMSE, Percent bias (PBIAS), and Correlation Coefficient (CC) metrics are used to evaluate the performance of the different methods. The daily scale satellite imputation results accurately captured rainfall variability more than statistical models. However, these results presented biases in extreme events. The higher complexity of the MLR models did not result in a significant improvement in model fit compared to the SLR. Both approaches were comparable on the monthly scale, although the statistical models poorly described seasonal variability. Consequently, the authors recommend applying bias corrections to optimize satellite estimates based on the findings. Moreover, they emphasize that the results cannot be easily extrapolated to other regions due to their dependence on climate and topography.

Demirhan and Renwick [45,58] suggested that statistical methods successfully impute missing values for different data types. They imputed horizontal solar irradiance time series with intervals from minutes to weeks in Australia. The authors compared various statistical methods, including linear interpolation, Stineman interpolation, Kalman filters with structural models, and Weighted Moving Averages (WMA). Measures of Relative Mean Absolute Error (RMAE), Relative Root Mean Square Error (RRMSE), and Mean Absolute Scaled Error (MASE) were used to compare the performance of the imputation methods. The results revealed that linear and Stineman interpolations, as well as Kalman filters, were highly accurate on hourly frequency series. On the other hand, the weighted moving average yielded outstanding results at both daily and weekly frequencies. This study emphasized the importance of selecting specific methods according to the data’s time-frequency characteristics, showing that simple techniques, such as interpolation, can be effective in high-frequency series. In contrast, more sophisticated statistical methods, such as the Kalman filter, offer clear benefits in a broader range of contexts.

Alternatively, Lai and Kuok [27] addressed the challenge of imputing missing data in rainfall series using Bayesian Principal Component Analysis (BPCA) in Kuching City, Malaysia. The performance of this technique was compared to the K-Nearest Neighbor Imputation (KNNI) method by evaluating metrics such as Bias, Efficiency, and RMSE. The results showed that the BPCA method presented considerable robustness and superior accuracy to the KNNI method, especially when data from nearby rainfall stations were incorporated as an additional reference. The work shows the advantage of the multivariate statistical approach (BPCA) over traditional univariate methods, particularly in contexts where the spatial correlation between neighboring stations is significant.

Nor et al. [29] compared several techniques for imputing missing data in daily rainfall series on the east coast of the Malaysian Peninsula. Data were obtained from 48 stations. The methods evaluated were the Replace by Mean algorithm, K-Nearest Neighbor (KNN), Markov Chain Monte Carlo (MCMC), Nonlinear Interactive Partial Least Squares (NIPALS), Random Forest (RF), and MLR. Performance evaluation was performed using RMSE, MAE, and NSE. The results showed that the Replace by Mean algorithm is the best method for single imputation. However, RF has demonstrated the best result when combined with MLR. Moreover, the data set is prone to the risk of changing the standard deviation and the data skewness when using the Replace by Mean method.

Alternatively, Azman et al. [25] developed a methodological comparison in the Lake Kenyir region, Malaysia, using classical and advanced statistical techniques to impute missing precipitation data. The methods analyzed included Expectation Maximization (EM), IDW interpolation, and Multiple Imputation (MI). The evaluation considered metrics such as RMSE, MAE, CC, and percent error ( % error). IDW interpolation performed better, with lower error values and higher correlation coefficients than MAE and MI. This work underlined the importance of IDW in contexts with limited spatial distribution of rainfall stations.

Wesonga [59] conducted multivariate imputation and forecasting of missing values in decadal wind speed data from Entebbe International Airport, Uganda. The methodology employed a fully statistical framework, utilizing the MICE technique within a Bayesian formulation, along with the MCMC method. After imputing approximately 28 % of missing records, the study applied an exponential smoothing model (Holt-Winters) to forecast future wind speed values. The results showed no statistically significant difference between the original and imputed datasets (p=0.6955), validating the reliability of the imputation. Moreover, the low smoothing parameter (α=0.014) indicated that recent observations had limited influence on the forecast, reflecting the high variability and stochastic nature of wind speed at the site.

Furthermore, Sattari et al. [60] evaluated various methods for imputing missing data in monthly precipitation records at six meteorological stations in southern Iran. The methods analyzed included classical statistical techniques such as Arithmetic Average (AA), Inverse Distance Weighted Method (IDWM), Normal Ratio method (NR), SLR, MLR, MI, NIPALS, and a method traditionally used by the United Kingdom (UK) Meteorological Office. This study also incorporated advanced data mining methods, such as decision trees (M5 Model Tree). Metrics used to evaluate the performance of these techniques included MAE, RMSE, Pearson's Correlation Coefficient (r), and Model Efficiency. The results indicated that advanced statistical methods, such as MLR, iterative algorithms (NIPALS), and MI, performed better compared to the others. Notably, MI yielded the most accurate results for data from highly correlated stations, as indicated by the lowest MAE and RMSE values and high correlation values.

Shtiliyanova et al. [37] focused on imputing missing data in air temperature time series, using a hybrid approach combining the ordinary classical geostatistical Kriging method with a Machine Learning-based procedure. This automated procedure was specifically applied to select and optimize model parameters using advanced statistical techniques, cross-validation, and machine learning based on historical data. The authors evaluated the method using daily and hourly temperature data from stations in various European climatic contexts, including France, Germany, Italy, the United Kingdom, and Switzerland. The results indicated that the hybrid approach performed efficiently for low and medium-altitude stations, particularly at hourly resolution, with high values of the NSE coefficient. However, accuracy decreased considerably at high-elevation stations (above 2000 m). The metrics used to evaluate performance were MAE, RMSE, Bias, and NSE, showing the effectiveness and flexibility of the hybrid method, especially when sufficient historical data are available to optimize model parameters.

On the other hand, Shabalala et al. [61] evaluated various methods for imputing daily maximum and minimum temperatures in the Limpopo province, South Africa. The techniques evaluated included AA, NR, IDW, Correlation Weighted (CW), Multiple Regression (MR), and the traditional UK method. The evaluation showed that MR and the traditional UK method achieved the best performance, with low MAE values (<1.8 °C for minimum and 1.0 °C for maximum temperatures) and high correlation coefficients (r>0.92). Furthermore, the metrics used were RMSE, Accuracy Ratio (AR), and Mean Bias Error (MBE).

Radi et al. [30] compared classical spatial interpolation and multiple imputation methods to fill missing data in daily rainfall series in Kuala Terengganu, Malaysia. The spatial methods evaluated were AA, NR, Inverse Distance (ID), CC, and MI. Moreover, they applied advanced statistical techniques, such as bootstrap multiple imputation, using the Amelia II method. All methods were evaluated with missing data at rates of 5 %, 10 %, and 20 %, resulting in only slight increases in MAE, accompanied by decreases in the SI and correlation coefficient. Overall performance remained stable despite the increase in missing data. The NR method and MI were the most suitable options, presenting better results in the three evaluation indices.

Similarly, Ismail and Ibrahim [26] compared several interpolation methods to impute missing daily rainfall and streamflow data at twelve stations in Terengganu, Malaysia. The methods evaluated included AA, NR, ID, and CC methods. In instances where the utilization of adjacent stations was not feasible, the historical mean of the same day and month in different years was employed. The evaluation of the methods was based on metrics such as RMSE, MAE, and CC under missing data percentages of 5 %, 10 %, 15 %, and 20 %. The results showed that ID was the most accurate method at several precipitation stations, while CC and NR proved superior for the river flow series. Generally, AA proved to be the least effective, exhibiting higher errors and lower correlations.

At the same time, Ghafarian Malamiri et al. [62] proposed the use of Singular Spectrum Analysis (SSA) and Multichannel Singular Spectrum Analysis (M-SSA) for the reconstruction of Land Surface Temperature (LST) series obtained by the MODIS satellite affected by clouds and outlier data. The study area encompassed areas of Iran, Turkmenistan, and part of the Caspian Sea, utilizing MOD11A1 products with a 1 km spatial resolution and daily frequency in 2015. The imputation process was based on decomposing the series into principal components through SSA. Subsequently, reconstruction of missing data was performed using spatiotemporal interpolation techniques. The imputation performance was evaluated using the RMSE, yielding an average value of 2.95 K compared to the original and reconstructed data. This work highlighted the effectiveness of SSA in reconstructing time series of climate variables with significant gaps, particularly in the context of satellite images affected by cloudiness.

Zhang et al. [34] proposed a model called Multiple Sine Function Decomposition (MSFD) for restoring missing data in monthly mean temperature time series in Guangzhou, China. This method is based on the successive decomposition of sine functions, a process that exploits the inherent cyclicity of climate data. The model reconstructs the missing data using a time series of successive approximations with sine functions. This reconstruction involves adjusting the model's parameters (amplitude, frequency, and phase) to meet specific criteria, such as achieving the desired level of accuracy or reaching a maximum number of iterations. Quantitative validation was performed by intentionally removing up to 48 consecutive points, resulting in reduced restoration errors (RMSE ≤ 2.21, SMAPE < 10 %, and MRE ≤ 0.1), even in the face of extensive gaps. This approach has been demonstrated to be effective in recovering cyclic data, yielding results that approximate real values, and overcoming the limitations of statistical methods that do not account seasonality.

In a study focused on the imputation of daily precipitation data in Portugal, Fagandini et al. [63] compared deterministic methods, such as the Food and Agriculture Organization (FAO) linear regression method, against geostatistical approaches, such as Ordinary Kriging (OK) and Simple Cokriging (SCK), the latter incorporating location elevation as a secondary variable. Monthly average semi-variograms were developed using data from 60 stations distributed in the Guadiana River basin to reduce the computational complexity of filling large volumes of missing data. Cross-validation revealed that OK outperformed the FAO method in terms of accuracy and that SCK did not significantly enhance the estimations. It was attributed to a limited correlation between elevation and daily precipitation in certain regions. The metrics used were ME, MAE, and RMSE, where OK provided a good balance between computational effort and estimation accuracy, considered the best estimator for the area under study.

Castello et al. [64] used recurrent neural networks for temperature prediction in Tungurahua, Ecuador. Data were obtained from the Mula Corral weather station. Although the primary objective was climate forecasting, the study also addressed the imputation of missing values using a linear interpolation method before model training. Three architectures were compared: Long-Short Term Memory (LSTM), Gated recurrent units (GRU), and Bidirectional Long-Short Term Memory (Bi-LSTM). The evaluations were performed using RMSE, MAE, MSE, and $R^{2}$. The results showed that the LSTM model performed better, with an RMSE value of 0.71. This result demonstrates that a straightforward approach, such as linear interpolation combined with deep networks, can be effective under low-loss conditions. This finding reinforces the potential of hybrid models where a previous statistical imputation allows the successful training of advanced Machine Learning architectures for forecasting weather variables.

Table 4 summarizes the features of each statistical or conventional method analyzed in this review. The computational complexity of each imputation method was systematically classified based on the dominant numerical operations, the dimensionality of the input data, and the empirical scalability observed in practical implementations. Each method was categorized into one of three distinct levels (Low, Medium, and High) based on the typical computational cost and scalability patterns identified through numerical analysis and algorithmic inspection[19].

• Low complexity encompasses methods whose runtime does not change (O(1)) or changes slowly (O(log(n))), regardless of the number of samples.
• Medium complexity includes methods whose computational demand scales linearly (O(n)) or semi-linearly (O(nlog(n))), characterized by direct analytical or single-pass calculations, minimal memory requirements, and negligible computational overhead as the data size increases.
• High complexity encompasses methods that exhibit predominantly polynomial ($O(n^{m})$) or exponential ($O(2^{n})$) computational scaling. These techniques involve intensive iterative computations, stochastic sampling, spectral decomposition, or inversion of large matrices, imposing significant computational demands that scale rapidly with dataset dimensionality and size.

**Table 4:** Summarization of the conventional methods presented in this review. Low complexity indicates algorithms with a computational runtime of O(1) or O(log(n)), medium complexity indicates algorithms with a linear O(n) or semi-linear O(nlog(n)) computational runtime, and high complexity indicates methods with a polynomial $O(n^{m})$ or exponential $O(2^{n})$ computational runtime.

Ref.	Method	Data type	Variable	Study area	Computational cost
[54]	DFT-IFFT	Climate variable	Temperature, humidity, wind speed	Ciénaga, Colombia	High
[28]	SLR	Climate variable	Rainfall	Kelantan, Malaysia	Low
[41]	PMM, midastouch	Climate variable	Solar radiation, wind speed, temperature, humidity	São Paulo, Brazil	High
[55]	AAM, GC, CCW, MCCW, MLR, IDW, MIDW, MCC-IDW, MLR	Climate variable	Precipitation	Upper Indus basin	Low, medium, high
[56]	VAR-IM	Climate variable	Temperature, wind, humidity, precipitation, solar radiation	Citeko, Indonesia	Medium
[57]	Median	Weather station	Air temperature, wind speed	Rosario Islands, Colombia	Low
[39]	SLR, MLR	Climate variable, Remote sensing	Precipitation	Central-west, Brazil	Low, medium
[45]	Linear and Stineman interpolation, WMA, Kalman filter	Climate variable	Solar irradiance	Australia	Low
[27]	BPCA	Climate variable	Rainfall	Kuching City, Malaysia	Medium
[29]	Mean, MCMC, MLR	Climate variable	Rainfall	East coast, Malaysia	Low, medium
[25]	IDW, MI	Climate variable	Rainfall	Lake Kenyir, Malaysia	High, medium
[59]	MICE (Bayesian, via MCMC)	Climate variable	Wind speed	Entebbe, Uganda	Medium
[60]	AA, NR, SLR, MLR, MI, NIPALS, IDW	Climate variable	Precipitation	Southern Iran	Low, medium, high
[37]	OK	Climate variable	Air temperature	Multiple (Europe)	High
[61]	AA, NR, CW, MR, IDW	Climate variable	Max & min temperature	Limpopo, South Africa	Low, medium, high
[30]	AA, NR, CC, MI, ID	Climate variable	Rainfall	Kuala Terengganu, Malaysia	Low, medium, high
[26]	AA, NR, CC, ID	Climate variable	Rainfall, streamflow	Terengganu, Malaysia	Low, high
[62]	SSA, M-SSA	Remote sensing	Land surface temperature	Iran, Turkmenistan, Caspian	High
[34]	MSFD	Climate variable	Mean temperature	Guangzhou, China	Low-medium
[63]	FAO regression, OK, SCK	Climate variable	Precipitation	Portugal	Low, high
[64]	Linear interpolation	Climate variable	Temperature	Tungurahua, Ecuador	Low

Complexity classifications were determined by analyzing the algorithmic structure described in the literature, with an emphasis on practical computational considerations rather than theoretical worst-case scenarios. Each method's complexity assignment provides clear guidance to researchers for selecting suitable imputation techniques based on available computational resources and specific dataset characteristics.

### Critical analysis for conventional methods

Conventional methods for imputing missing values in climate time series vary in performance depending on data characteristics, such as spatial and temporal resolution, inter-variable correlation, and the extent of missingness. Interpolation methods are computationally efficient and suitable for short gaps or series with smooth or periodic behavior [45,54,64]. However, they often fail when gaps are significant or trends are nonlinear, assuming continuity.

Among spatial interpolation techniques, the IDW method and its variants (e.g., MIDW, MCC-IDW) were frequently employed in the reviewed studies due to their ease of implementation and acceptable accuracy in moderately dense station networks. Nevertheless, IDW incorporates distance-based weighting from multiple stations, making it more computationally intensive than univariate interpolation, primarily when used with high-resolution spatial grids or extensive observation networks [25,55,60,61].

Other model-based techniques include pure regression models (SLR, MLR), semi-parametric approaches (PMM), and multivariate probabilistic models such (as BPCA). These methods exploit inter-variable correlations to estimate missing values and have shown reliable performance in multivariate contexts [27,28,39]. Nonetheless, their effectiveness diminishes in the presence of weak correlations or incomplete auxiliary data.

Geostatistical approaches, such as Kriging and Cokriging, exploit spatial structure and perform well in dense monitoring networks [63]. Despite their accuracy, these methods are computationally intensive compared to other conventional methods and require modeling spatial covariance, which limits their use in data-scarce or resource-limited environments, particularly when machine learning (ML) techniques are used to optimize model parameters, as addressed in [37].

Simple averaging methods (e.g., mean, median) remain popular due to their ease of use and low computational cost [29,57]. However, they tend to distort the statistical properties of the series and underestimate variability. The choice of method must consider the data structure, the pattern of missingness, and computational constraints. While simple methods may suffice for initial analysis, complex datasets require more robust or hybrid imputation strategies.

### Machine learning-based methods

**Fig. 4:**
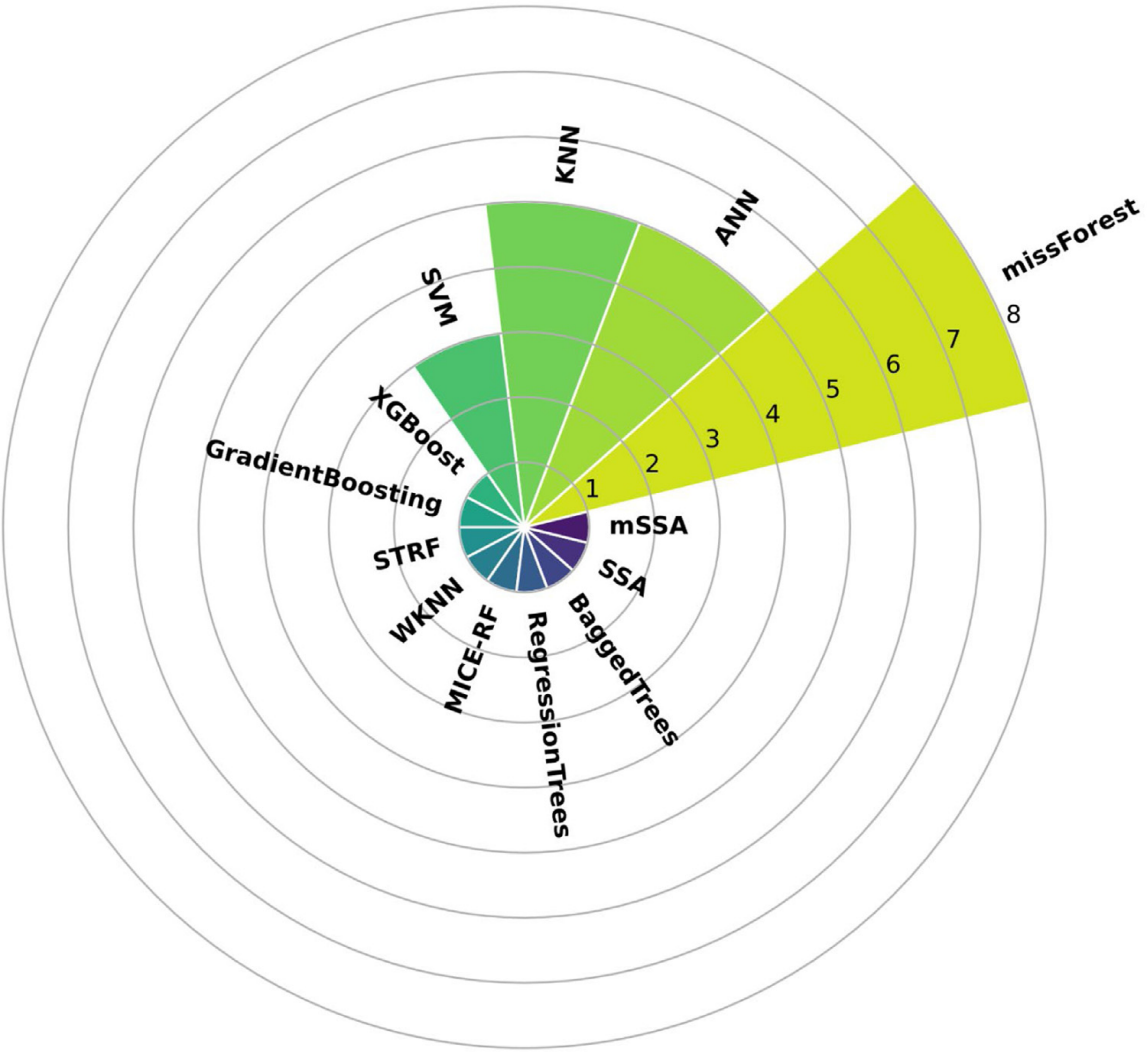
Frequency graph of the most used machine learning methods by the works found in our methodology.

Machine learning algorithms are one of the most widely used techniques for handling missing data due to their ability to recognize patterns in datasets where conventional methods are limited. Fig. 4 shows a frequency graph with the machine learning methods found under the methodology described above.

This figure shows that the most used methods are missForest, KNN, and Artificial Neural Networks (ANN). The missForest method was proposed by Stekhoven and Bühlmann [65] for handling missing data in mixed datasets. This method has been used in several works as a benchmark to compare against other techniques, including conventional ones. For example, Addi et al. [66] conducted a comparative study among 12 statistical and machine learning methods applied to rainfall time series in Ghana's Pra and Densu river basins. The metrics used to evaluate their performance were RMSE, MAE, Bias, $R^{2}$, SI, NSE, and Kolmogorov-Smirnov performance statistics. The results suggest that regression techniques, Probabilistic Principal Component Analysis (PPCA), and missForest were the techniques that obtained the lowest and most competitive errors. These methods provide a more accurate estimation of the number of dry and wet periods and moderate to extreme rainfall values.

On the other hand, Vidal-Paz et al. [67] compared several methods, including missForest, for handling missing precipitation time series data in Galicia, Spain. To evaluate this comparison, they used four established metrics to assess the accuracy of the predicted data against the actual data. These metrics were MAE, Relative Error (RE), RMSE, and NRMSE. In this work, missForest was the method that obtained the most competitive results, i.e., with the lowest percentage error in estimating missing data.

Similarly, Qaraghuli et al. [68] performed a comparison of several machine learning methods (univariate and multivariate), including missForest. They aimed to impute missing data in precipitation, temperature, and relative humidity time series obtained from the Mosul station in Iraq, spanning the period from 1980 to 2013. RMSE and Kling-Gupta Efficiency (KGE) metrics were used to evaluate and compare the performance of each method. Based on these metrics, seasonal decomposition proved to be the most effective method for univariate data. In contrast, the most competitive method for multivariate data sets was KNN for precipitation time series, the *norm.predict* algorithm for temperature time series and missForest for relative humidity time series.

In a recent study, Ismail et al. [69] compared two machine learning methods (KNN and missForest) and a statistical method (MICE) for handling missing precipitation time series data in two Moroccan regions: the Moulouya basin and the Sous Massa basin. MAE, RMSE, Coefficient of Variation of RMSE (CVRMSE), and NRMSE were used to compare the performance of the models. The results suggested that missForest was the method with the most competitive efficiency, followed by MICE and KNN.

Yashas and Varija [70] proposed a two-step methodology to fill in missing temperature data from four stations (Karwar, Honnavara, Shirali, and Mangalore) in coastal Karnataka, India. The first step of this methodology includes the SSA method to fill in the small sporadic gaps. Meanwhile, the second step includes multivariate techniques (IDW, Kriging, Spatial Regression Test (SRT), Point Estimation method of Biased Sentinel Hospital-based Area Disease Estimation (P-BSHADE), RF, Support Vector Machines (SVM), and missForest) to fill in longer gaps. The evaluation metrics used were MBE, MAE, RMSE, Mean Absolute Relative Error (MARE), standard deviation of the residual, RMSRE, Maximum absolute relative error (erMAX), NSE, SI, Legates's Coefficient of Efficiency (LCE), Kolmogorov-Smirnov performance statistics, Parameter OVER, Combined Performance Index (CPI), and Taylor diagram. The results suggest that SSA obtains competitive results for filling sporadic small gaps. In contrast, for maximum temperatures, P-BSHADE and SVM obtain superior results, which is attributed to their ability to capture spatial and/or temporal heterogeneity.

Finally, Wang et al. [71] applied the Spatial-Temporal Random Forest (STRF) method to fill gaps in Landsat LST images caused by cloud cover in the USA. The authors applied experiments using data from six regions with different land covers. RMSE and CC were employed as evaluation metrics. The findings demonstrate that this method exhibits satisfactory accuracy compared to alternative approaches. The RMSE and CC metrics facilitate a quantitative assessment of the accuracy of the aforementioned method.

Regarding the use of Random Forest, Kannegowda et al. [72] compared this method against univariate and multivariate methods of varying length to fill in missing precipitation data in the tropical humid region of Kozhikode, Kerala, India. RMSE, MAE, NSE, and MARE metrics were used for their evaluation. The results suggest that the connected Kalman Smoothing (KS) Models are more competitive against the compared methods for univariate time series. Meanwhile, Principal Component Analysis (PCA) and RF outperform the other methods for multivariate series. These methods even improve the imputation of large data spaces compared to univariate methods, which are limited to small missing data spaces.

Similarly, Jing et al. [31] employed the RF model combined with the MICE method (MICE-RF) to develop a tool for filling in missing data from multiple observational variables. Hydrometeorological data (evaporation, mean surface temperature, precipitation, atmospheric pressure, relative humidity, duration of light alone, wind speed, and wind direction) obtained from Hanzhong Station, Hanjiang River Basin, China, were employed in this work. MAE, RMSE, and NSE metrics were used to evaluate the performance of the proposal. The results suggest that MICE-RF achieves the most competitive accuracy for filling in missing data compared to classical techniques (traditional linear imputation, mean imputation, spline imputation, and KNN), making it a viable option for handling climatic missing data.

Another work where the RF method was used was introduced by Kane et al. [73]. This research presents a comparative study of various missing data imputation techniques applied to climatic time series. Data were obtained from the Sudanese Zone in West Africa. Moreover, the climate variables used were Meteorological Temperature, Solar Radiation, Swin, Swout, Rnet, Humidity, Vapor Pressure Deficit, Latent Heat Flux, Soil Moisture, Soil Temperature, and Carbon Flux. Three methods were evaluated: SSA for univariate series, M-SSA for multivariate series, RF, and MLR models, the latter two using MICE strategies. MSE and MAE metrics were used to measure their performance. The results showed that SSA finds competitive behavior in capturing patterns for independent variables, while M-SSA excels with highly correlated features. Even though M-SSA processes data at a faster rate, SSA remains the preferred option in the majority of cases. SSA consistently outperforms competing models when most sensors do not record data concurrently. The imputation accuracy was affected by problems posed by long missing segments.

Concerning Artificial Neural Networks, Niyazi et al. [74] compared several missing data imputation techniques to estimate rainfall time series extracted from eight meteorological stations located near Al-Madinah Al-Munawarah City, Saudi Arabia, over a five-year period, using two of them as test data. The techniques evaluated were AA, IDW, NR, Satellite Products, Tropical Rainfall Measuring Mission (TRMM), Integrated Multi-satellitE Retrievals for GPM (IMERG-GPM), CHIRPS, MERRA-2, and artificial intelligence-based and Feed-Forward Backpropagation Neural Network (FFBP-NN). The results indicated that FFBP-NN achieved the highest correlation values, outperforming the compared methods.

Sanhudo et al. [42] introduced a machine learning methodology that applies regression algorithms to rectify erroneous values in the datasets. Moreover, their proposal groups weather stations based on recorded weather conditions to improve the regression models. This methodology uses the k-medoids algorithm and Dynamic Time Warping (DTW) as a similarity measure. ANN and Support Vector Regression (SVR) models are evaluated as exemplary regression algorithms with different sets of predictors. The MSE is used as a performance metric. This analysis examines variables such as atmospheric pressure, last-hour maximum and minimum atmospheric pressure, solar radiation, air temperature, dew point temperature, last-hour maximum and minimum temperature, last-hour maximum and minimum dew point temperature, relative humidity, and last-hour maximum and minimum relative humidity recorded in Brazil. The results indicate that ANN slightly outperforms SVR in predicting the weather variable studied, making the machine learning-based methods competitive for time series imputation.

Saubhagya et al. [75] proposed a Multilayer Neural Network Perceptron (MLP), which captures temporal variability in rainfall series and learns patterns in the data over time. Moreover, they employed the Spatial Kriging method to capture the spatial relationships between measurement stations and estimate missing values, taking into account the geographic proximity and correlation between stations. The MAE, RMSE, and $R^{2}$ metrics were used for their evaluation. The results suggest that smaller values of MAE and RMSE and larger values of $R^{2}$ indicate better forecasting. Therefore, the hybrid model achieved the best performance compared to the other methods.

Canchala-Nastar et al. [76] implemented an approach for handling missing data in monthly precipitation time series obtained from 45 rainfall stations in southeastern Colombia. This approach employs a nonlinear generalization of the standard principal component analysis method, utilizing an ANN known as Nonlinear Principal Component Analysis (NLPCA). The results suggest that NLPCA is a robust methodology for imputing missing precipitation data.

Furthermore, Lima et al. [40] proposed a two-phase methodology to impute climate series data in ten different regions of Brazil. The first phase of the methodology includes triangulation methods such as AA, IDW, MIDW, NRM, and Regional Weight (RW). Machine learning techniques, including ANN, SVM, Regression Trees (RT), and Bagged Trees (BT) were employed for the second phase. For the evaluation of the imputation methods, we used NRMSE and the ANOVA statistical test. The results indicated that the best configuration involved a widely used mathematical triangulation model combined with neural networks. This combination yielded the most satisfactory results in predicting missing data, outperforming traditional triangulation methods.

On the other hand, techniques such as Self-Organizing Maps (SOM), a type of ANN, have also been explored. Nkiaka et al. [77] employed SOMs to fill rainfall time series data in the Logone River basin, which spans Cameroon, the Central African Republic, and Chad. The results were evaluated using $R^{2}$ and mean topographic error. These results suggest that SOMs are a robust and efficient method for treating missing data in hydrometeorological time series.

Another popular method for imputing missing data in weather time series is KNN, which is also referenced as a comparison method against other proposals. Oriani et al. [46] proposed two approaches based on historical data patterns for estimating incomplete data segments. These approaches were an iterative version of KNN (IKNN) and a new algorithm called Vector Sampling (VS) that combines concepts of multiple point statistics and resampling. The data used were daily precipitation time series obtained from five regions in Denmark, Australia, and Switzerland. The results were evaluated using RMSE and BIAS metrics, suggesting that the behavior of each tested algorithm will depend on the soil characteristics. For example, the authors found that, in flat terrain with spatially homogeneous rainfall events, geostatistical interpolation tends to minimize the mean error. In contrast, in mountainous regions with non-stationary rainfall statistics, machine learning can better recover rainfall patterns. Finally, they concluded that the VS algorithm requires minimal parameterization and is a convenient option for routine application in complex and poorly gauged terrain.

Aieb et al. [78] proposed a new imputation algorithm that optimizes several regression methods, including Hot-Deck, KNNI, Weighted K-Nearest-Neighbors Imputation (WKNNI), MI, linear regression, and the simple mean method. This algorithm was tested using precipitation time series data from the Soummam basin in Algeria, and evaluated based on $R^{2}$, adjusted $R^{2}$, RMSE, and MAE. The results suggest that the Hot-Deck, KNNI, and WKNNI methods obtain competitive results in missing data imputation, regardless of the percentage of missing data.

In addition to the methods previously discussed, gradient-boosting techniques have been employed to handle missing data. For example, Körner et al. [79] used the Gradient Boosting (GB) method to fill missing or erroneous data gaps in meteorological time series. In this case, they utilize an hourly time series dataset of air temperature, wind speed, and relative humidity for station-based observations in Germany. They apply the MAE, RMSE, and $R^{2}$ models as evaluation metrics. The analysis reveals that GB yields minor errors when estimating missing values with a median RMSE. On the other hand, the comparison between the results of GB and other gap-filling techniques, such as neural networks or multiple linear regression, shows considerably better statistics. Moreover, this comparison demonstrates that the GB approach outperforms the other techniques, particularly in terms of computational time, performance, and handling of missing data values.

Similarly, Başakın et al. [80] introduced a method that combines the eXtreme Gradient Boosting (XGBoost) algorithm with the Differential Evolution (DE) algorithm, referred to as XGBoost-DE. This method imputes missing data found in solar radiation measurements, which is a crucial meteorological variable in terms of climate dynamics and energy technologies. These data were obtained in the closed basin of Konya, Turkey. The results were evaluated using NSE and KGE, revealing that the XGBoost-DE model obtained competitive values in all the defined missing segments.

Table 5 summarizes the machine learning-based methods discussed in this section. The table structure and definitions of the computational complexity categories are similar to those of conventional methods.

**Table 5:** Summarization of the machine learning-based methods presented in this review. Low complexity indicates algorithms with a computational runtime of O(1) or O(log(n)), medium complexity indicates algorithms with a linear O(n) or semi-linear O(nlog(n)) computational runtime, and high complexity indicates methods with a polynomial $O(n^{m})$ or exponential $O(2^{n})$ computational runtime.

Ref.	Method	Data type	Variable	Study area	Computational cost
[66]	missForest, KNN, ANN	Climate variable	Rainfall	Pra and Densu river basins, Ghana	Medium, medium, high
[67]	missForest	Climate variable	Rainfall	Galicia, Spain	Medium
[68]	KNN, missForest	Climate variable	Precipitation, temperature, and relative humidity.	Mosul station, Iraq	Medium, medium
[69]	KNN, missForest	Climate variable	Precipitation	Moulouya and Sous Massa basins	Medium, medium
[70]	RF, SVM, missForest	Climate variable	Daily maximum and minimum temperature	Karnataka, India	Medium, high, medium
[71]	STRF	Remote sensing	Land surface temperature (LST)	USA	Medium
[72]	RF	Climate variable	Rainfall	Kozhikode, Kerala, India	Medium
[31]	MICE-RF	Climate variable	Evaporation, mean surface temperature, precipitation, atmospheric pressure, relative humidity, duration of light alone, wind speed, and wind direction	Hanzhong Station, Hanjiang River Basin, China	Medium
[73]	RF	Climate variable	Meteorological temperature, solar radiation, swin, swout, Rnet, humidity, vapor pressure deficit, latent heat flux, soil moisture, soil temperature, and carbon flux	Sudanese Zone in West Africa	Medium
[74]	FFBP-NN	Climate variable	Rainfall	Al-Madinah Al-Munawarah city, Saudi Arabia	High
[42]	ANN, SVR	Climate variable	Atmospheric pressure, last hour maximum and minimum atmospheric pressure, solar radiation, air temperature, dew point temperature, last hour maximum and minimum temperature, last hour maximum and minimum dew point temperature, relative humidity, and last hour maximum and minimum relative humidity	Brazil	High, high
[75]	MLP	Climate variable	Rainfall	Ratnapura Area, Sri Lanka	High
[76]	NLPCA	Climate variable	Rainfall	Colombia	High
[40]	ANN, SVM, RT, BT	Climate variable	Daily maximum temperature, average, monthly maximum temperature, altitude, latitude, longitude and timestamp.	Brazil	High, high, medium, medium
[77]	SOM	Climate variable	Rainfall	Cameroon, the Central African Republic, and Chad	High
[46]	IKNN	Climate variable	Daily precipitation	Denmark, Australia, and Switzerland	Medium
[78]	KNNI, WKNNI	Climate variable	Precipitation	Soummam basin, Algeria	Medium
[79]	GB	Climate variable	Air temperature, wind speed, and relative humidity	Germany	Medium
[80]	XGBoost-DE	Climate variable	Solar radiation	Konya, Turkey	High

### Critical analysis for machine learning-based methods

Unlike conventional methods, most machine learning algorithms exhibit medium to high computational complexity, meaning they typically require more time and computational resources for execution. Particularly, missForest is the method most widely used for imputing climate data with missing values [31,[66], [67], [68], [69], [70], [71], [72]–,^73^], yielding the most robust results in all cases, especially in data with a large gap of missing values [72]. However, the main disadvantage of this method is its computational complexity, which increases with the sample size. Moreover, RF, combined with statistical methods such as MICE-RF [31], has demonstrated competitive performance in the areas where it has been employed. However, MICE-RF could be affected by non-correlated variables.

Other methods, such as the KNN method, exhibited variable behavior depending on the climatology of the study area, the geographical station, and the variable employed, among other factors [46,66,68,69,78]. However, it is the most commonly employed method for comparing the results of new imputation techniques due to its simplicity and ease of implementation. Moreover, KNN can be affected by the sample size, with its performance decreasing as the sample size increases.

ANNs are still widely employed for imputing climate missing data. However, most authors demonstrated that, when combined with other techniques, such as triangulation methods [40] and statistical methods [75,76], ANNs yielded more competitive results compared to other methods or even when used separately. Moreover, when ANNs were compared with other machine learning-based methods, such as SVM [40,42], the performance of ANNs was superior to that of the other methods in most cases [42,74]. Furthermore, the main disadvantage of ANNs is the higher computational cost associated with their performance, which is particularly noticeable when a large climate time series is employed to impute missing values.

Similarly, SOMs were employed to impute missing values in climate time series [77]. However, their performance is reduced with larger gaps. Moreover, SOMs require a sufficiently large amount of data to be trained, which entails a high computational cost.

On the other hand, GB demonstrated superior performance than ANNs with a lower computational cost [79]. However, it is considered a black box, showing no relationship between the predictors and the response. Moreover, its computational cost can be affected by the sample size, like the aforementioned methods. Therefore, improved versions of GB have been proposed, such as XGBoost-DE, which optimizes its hyperparameters using DE [80]. Furthermore, DE converts this method into a more complex technique, increasing its computational cost.

It is essential to note that the performance of each machine learning-based method used for imputing missing values in climate time series is influenced by both the calibration of its parameters and the characteristics of the datasets (close meteorological stations, study area geographical location, time series size, etc.). Therefore, it is necessary to analyze the data of each study case to decide the method to employ. Moreover, an extensive analysis of the parameter values used in each technique is necessary to ensure that the chosen configuration is suitable for the problem.

### Deep learning-based methods

**Fig. 5:**
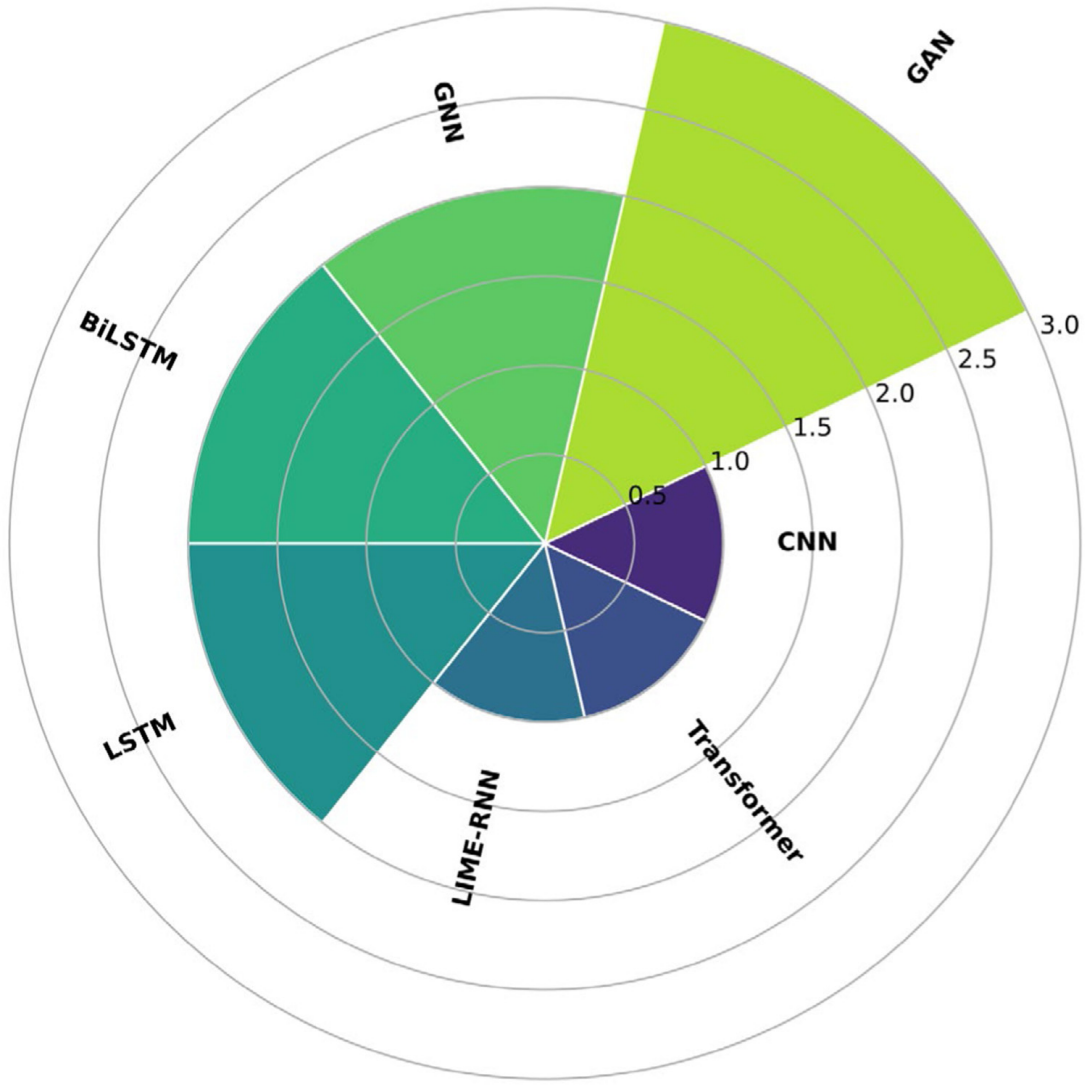
Frequency of the deep learning methods most frequently used in the papers found in this state-of-the-art review.

Concerning methods based on deep learning, Fig. 5 shows the frequency of techniques used to impute missing data in climate series. Dimitri et al. [35] used a Graph Neural Network (GNN) for imputing missing values in climate time series. Moreover, data were extracted from weather stations with new technologies that are crucial for studying climate prediction, ecosystems, and agricultural management. The study was conducted with five sensor nodes deployed on a farm in Siena, Italy. The climate variables employed were the temperature (in degrees Celsius, °C), relative humidity (percentage of humidity in the air), solar radiation intensity (measured as a function of light sensor resistance), leaf wetness (ratio of water present on plant leaves), and liquid precipitation (measured by rain buckets installed on the advanced nodes). The evaluation metrics used in the study were RMSE and MAE. The results suggest that the Graph Neural Network (GNN) successfully addressed missing data imputations in their meteorological dataset.

On the other hand, Yang et al. [32] proposed an adversarial generative learning framework for temperature time series imputation under the condition of observed data. They employ a modified bidirectional Recurrent Neural Network (RNN) structure as the G-generator to impute the missing values. Moreover, they conducted imputation and classification experiments on several real-world time series data sets for information verification. The MAE metric was employed to verify their evaluation. The experimental results reveal a significant difference between the proposal and the most recent models. Consequently, the proposal is considered a suitable model for applications involving missing data in climate time series.

Similarly, Popolizio et al. [36] proposed a Generative Antagonistic Imputation Antagonistic Network (GAIN) to address the problem of incomplete time series in meteorological data. The method is used to complete a set of high-frequency time series temperature variables. It involves a generator that imputes missing data and a discriminator that distinguishes between observed and imputed values. The research used temperature values from 98 meteorological stations in Apulia, Italy. Performance was evaluated using the RMSE. Finally, the authors concluded that the temperature time series obtained by imputing missing values using GAIN is very close to the real data in terms of RMSE. Therefore, it is a promising method for imputing missing data in meteorological time series.

Xie et al. [33] employed Bi-LSTM to fill in large intervals of missing data in high and low-frequency temperature meteorological records. High-frequency data was recorded every half hour. Meanwhile, the low-frequency temperature was obtained manually (morning, noon, and night). The data were obtained from the Dinghushan National Forest Ecosystem Scientific Observation and Research Station in Guangzhou, China. Unlike other methods, the model achieves higher accuracy and better generalization capability, even with lags of up to 60 days. They also applied the RMSE as an evaluation metric.

Kulanuwat et al. [81] utilized two methods to identify values that deviate from standard patterns in hydrometeorological time series. Those methods include Median Absolute Deviation (MAD), which utilizes a moving window technique for outlier detection, and Bi-LSTM for imputing missing data. The data were obtained from telemetry stations throughout Thailand. The F1-score and RMSE metrics indicate that the proposed methods demonstrate desirable performance suitable for real-time detection. The methods with the best results were the median-based statistical approaches for anomaly detection, the linear method, and the spline method for data imputation. These methods provided promising results for non-cyclic data behaviors. On the other hand, Bi-LSTM performed relatively well on time series data with strong seasonality and drastic changes in a short period.

Moreover, Ma et al. [82] proposed a methodology for handling missing data in various data sets, including temperature time series. This methodology employed data from England between January 1723 and December 1970. The method employed in this work was the Linear Memory Vector Recurrent Neural Network (LIME-RNN). LIME-RNN is a RNN with a learned linear combination of previous historical states. LIME-RNN was compared against several state-of-the-art methods for missing value imputation. Based on RMSE and MAE metrics, the approach can handle the imputation of randomly distributed and consecutive missing data. Additionally, LIME-RNN enables imputation and prediction when forecasting time series with missing values.

On the other hand, Wang et al. [83] developed an innovative transformer-based deep learning model to fill data gaps in temperature, precipitation, and evapotranspiration variables. This model incorporates a self-attenuation mechanism using causal convolution, which allows the neural network to capture the local context of the Gravity Recovery and Climate Experiment (GRACE) time series data. The evaluation metrics used were NSE and RMSE. This study has produced a reliable gap-filling product that addresses 11-month data gaps and 24 isolated gaps, ensuring the continuity of GRACE data for various academic applications.

Ngoc Tran et al. [38] applied a deep learning methodology to reconstruct missing data from a dataset extracted in the Northern Hemisphere. They also use evaluation metrics such as RMSE, $R^{2}$, KGE, and Mean Error (ME). The data used in this study were air temperature, snow depth, and ground temperature from the northern hemisphere high latitudes, arctic and subarctic regions, North America, Europe, Asia, and Greenland. The article concludes that the effectiveness of using deep learning and ERA5-Land reanalysis data lies in their ability to reconstruct missing data in the time series of the aforementioned variables. Notably, this approach generated an observed-reconstructed database that enables the analysis of the dynamics and relationships between these variables. This finding contributes to obtaining more efficient data with greater accuracy.

Viana et al. [84] utilized Generative Adversarial Networks (GAN) for imputing missing data on average temperature, wind direction, average wind speed, maximum instantaneous wind speed, and solar radiation extracted from the Quinta de Santa Bárbara and Pinhão regions in Portugal. GANs were compared against a well-known model, simple linear regression. The results suggest that GANs yield acceptable values when processing wind speed and solar radiation time series, whereas they encounter incongruent missing values in the other climatic variables. The authors suggest that further studies and research are needed to improve the performance of GANs on this type of data.

Another work, where the missing values of several climatic variables are estimated, is presented by Boujoudar et al. [85]. The imputation of the variables temperature, precipitation, humidity, direct normal irradiation, global horizontal irradiation, diffuse horizontal irradiation, wind speed, and direction is performed. MLP, LSTM, and Convolutional Neural Networks (CNN) were evaluated using RMSE, MAE, and $R^{2}$ metrics. The results suggest that LSTM and CNNs outperform regarding short-term missing data gaps, while MLP outperforms the other methods on 3-day missing segments. The authors conclude that the choice of model for imputing missing data depends on the specific gap size, with CNNs excelling for missing gaps of one month.

Finally, Decorte et al. [86] propose a hybrid approach that utilizes 12 different methods for imputing missing data. In this work, three different types of approaches were employed. These approaches were categorized into Time-Based Imputation, Spatial Correlation-Based Imputation, and Hybrid (Spatial + Temporal) Imputation. The Time-Based Imputation includes the Mean Imputation and Spline Interpolation methods. The Spatial Correlation-Based Imputation includes KNN, MICE, Markov Chain Monte Carlo (MCMC), and MissForest. Finally, the Hybrid Imputation includes k-nearest Neighbor Estimation (AKE), Data Estimation using Statistical Models (DESM), Matrix Completion (MC), Multiple Imputation using Denoising Autoencoders (MIDA), Bidirectional Recurrent Imputation for Time Series (BRITS), and Multi-directional Recurrent Neural Network (M-RNN). The data used to compare each method were temperature and soil moisture time series from the Flanders region, Belgium. The results were evaluated using RMSE, MAE, and Prediction Coverage Error metrics, suggesting that the MC method outperformed the other methods for all types of missing data. On the other hand, deep learning-based methods performed poorly for both missing patterns, which may be attributed to the characteristics of the dataset. The authors conclude that the methods exploiting spatial correlations within the dataset perform competitively compared to the other methods.

Like the previous sections, Table 6 describes the primary features of the aforementioned methods.

**Table 6:** Summarization of the deep learning-based methods presented in this review. Low complexity indicates algorithms with a computational runtime of O(1) or O(log(n)), medium complexity indicates algorithms with a linear O(n) or semi-linear O(n log(n)) computational runtime, and high complexity indicates methods with a polynomial O(n^m) or exponential O(2^n) computational runtime.

Ref.	Method	Data Type	Variable	Study area	Computational cost
[32]	RNN	Climate variable	Time series	China	High
[36]	GAN	Meteorological variable	Temperature	Apulia, Italy	High
[33]	BiLSTM	Meteorological variable	High-frequency temperature and low-frequency temperature	Guangzhou, China	High
[81]	BiLSTM	Hydrometeorological variable	Water level	Thailand	High
[82]	LIME-RNN	Climate variable	Temperature	England	High
[83]	Transformer	Climate variable	Temperature, precipitation and evapotranspiration	Global scale	High
[38]	LSTM	Climate variable	Air temperature, snow depth, ground temperature	Northern Hemisphere, covering regions such as: Arctic and Sub-Arctic regions North America Europe Asia Greenland	High
[84]	GANs	Climate variable	Temperature, wind direction, average wind speed, maximum instantaneous wind speed and solar radiation	Quinta de Santa Bárbara and the Pinhao region, Portugal	High
[85]	MLP, LSTM and CNNs	Meteorological variable	Temperature, precipitation, humidity, direct normal irradiation, global horizontal irradiation, diffuse horizontal irradiation, wind speed, and direction	Green Energy Park facility in BenGuerir, Morocco,	High
[86]	RNN	Climate variable	Temperature and humidity	Flanders, Belgium	High

### Critical analysis for deep learning-based methods

Deep Learning methods represent a significant advance in artificial intelligence, as evidenced by their competitive results in the problems where they have been tested. Methods such as RNN, LSTM, BiLSTM, GANN, LIME-RNN, Transformers, GANs, MLP, and CNNs represent different approaches to modeling complex data.

Considering all the models previously used and visualizing them on a scale from the lowest to the highest, the following are highlighted. Methods such as MLPs, although considered easy to implement and simple in terms of their structure, are limited in handling special or complex tasks, resulting in a lower level than that of other models. On the other hand, CNNs are very effective due to their ability to capture data, although more complex than the MLP model. CNNs are a good choice for medium-complexity datasets [85].

In general, RNNs are considered a recommendable option for handling sequential data. Some studies show that, for multivariate time series, RNNs outperform MLPs in the time series imputation task. Although it is a valuable and stable model, it has limitations, such as the difficulty in defining proper parameter settings for the training process and the inability to process long sequences when the tanh and RELU functions are used as the activation function [32,87]. As a consequence, RNNs may be unsuitable for processing significant data gaps.

On the other hand, it is noted that the LIME-RNN method enables both imputation and prediction tasks to be performed simultaneously. However, demonstrating the effectiveness of the model through interpretation in sequential models requires a more specific analysis. In terms of its results, this model surpasses most existing time series imputation methods [82]. However, similar to RNNs, the main disadvantage is its computational cost, which increases as the architecture and data become more complex.

GANs and their variants excel at generating realistic data, but they also face stability challenges. Likewise, in the case of the Transformers method, it is considered that they outperform RNNs in terms of their capacity to handle long-term missing data and are similar to methods such as LIME-RNN and GANs in terms of computational complexity. However, Transformers are considered more computationally expensive models [84].

In summary, it is worth noting that although these methods demonstrate competitive or even efficient results, they incur a high computational cost (see Tables 4, 5, and 6). Therefore, conventional and machine learning-based techniques are still employed for imputing climate time series within the scientific community.

### New research areas for data missing imputation in climate time series

Based on the findings of this review, the following future research opportunities are identified for handling missing data in climate time series:

1. *Climate indices time series reconstruction.* This analysis demonstrates that the most employed climatic information used for reconstructing time series is direct variables rather than climate indices. However, these indices help to describe phenomena such as drought, which needs both direct variables and climate indices to characterize its behavior.
2. *Research in North and Central America.* According to the review findings, research on missing data handling in climate time series has been limited to South America, excluding the countries of Central and North America. For example, the United States recorded one research study. At the same time, countries such as Mexico, where climate monitoring networks experiencing increasing difficulties, are not involved in this type of research.
3. *New proposals for hybrid algorithms or ensembles.* We propose combining different methods to reconstruct missing data based on our results. A unique method can perform optimally only for certain variables but not for others; even this performance may vary for different regions.
4. *Imputation of satellite time series data.* Although the temporality of satellite information is constant, i.e., data is always recorded, the missing information is due to the presence of clouds. This situation involves missing data in satellite information that requires imputation. Research in this scenario is limited, suggesting that it may be an area of interest for future studies.

## Conclusion

The imputation of missing data in a time series of climate variables is a key challenge to accurate climate monitoring and mitigation strategy. This review synthesizes the current status of imputation techniques, including conventional, ML, and DL methods, applied to various climate variables and study areas. ML and DL methods, particularly RF, RNNs, and hybrid techniques, perform significantly better in multiple scenarios with missing data that exhibit spatial and temporal correlations. However, conventional statistical methods remain relevant because of their low computational cost and effectiveness in contexts with infrequent missing data and scenarios where missing values do not occur in long sequences. Temperature, precipitation, and humidity were the most frequently observed climate variables. On the other hand, the limited attention to the reconstruction of climate indices stands out as a line of future research. The geographic distribution of research reveals a notable asymmetry, with a concentration in Asia and Europe. Conversely, North America and Central America, notably Mexico, have not reported significant work despite their vulnerability to extreme weather events. On the other hand, satellite data have been little exploited due to the presence of clouds, so future research should focus on overcoming these limitations. Finally, based on the results, a combination of different imputation methods is proposed since their optimal performance varies according to the variable type and may not be generalized to different regions.

## Ethics statements

Not applicable

## CRediT authorship contribution statement

**Lizette Elena Alejo-Sanchez:** Methodology, Writing – original draft. **Aldo Márquez-Grajales:** Conceptualization, Methodology, Writing – original draft. **Fernando Salas-Martínez:** Conceptualization, Methodology, Writing – original draft. **Anilu Franco-Arcega:** Supervision, Writing – review & editing. **Virgilio López-Morales:** Supervision, Writing – review & editing. **Otilio Arturo Acevedo-Sandoval:** Supervision, Writing – review & editing. **César Abelardo González-Ramírez:** Supervision, Writing – review & editing. **Ramiro Villegas-Vega:** Conceptualization, Methodology, Writing – original draft.

## Declaration of competing interest

The authors declare that they have no known competing financial interests or personal relationships that could have appeared to influence the work reported in this paper.
